# Supplementary material for: OpenMM 7: Rapid development of high performance algorithms for molecular dynamics
Source: PLoS Comput Biol. 2017 Jul 26;13(7):e1005659. doi: 10.1371/journal.pcbi.1005659 (PMC5549999; doi:10.1371/journal.pcbi.1005659)
Supplement: S1 Source Code — (ZIP) [file pcbi.1005659.s002.zip › OpenMM-7.0.1-Source/docs/api-python/generated/simtk.openmm.openmm.AmoebaStretchBendForce.html]

AmoebaStretchBendForce — OpenMM 7.0.1.dev-5e86c4f documentation


# AmoebaStretchBendForce¶

*class* `simtk.openmm.openmm.``AmoebaStretchBendForce`(*\*args*)¶
:   This class implements the Amoeba stretch-bend interaction.

    To use it, create a StretchBendForce object then call addStretchBend() once for each stretch-bend. After a stretch-bend has been added, you can modify its force field parameters by calling setStretchBendParameters(). This will have no effect on Contexts that already exist unless you call updateParametersInContext().

    `__init__`(*self*) → AmoebaStretchBendForce¶
    :   \_\_init\_\_(self, other) -> AmoebaStretchBendForce

        Create an AmoebaStretchBendForce.

    Methods

    |  |  |
    | --- | --- |
    | `__init__`((self) -> AmoebaStretchBendForce) | \_\_init\_\_(self, other) -> AmoebaStretchBendForce |
    | `addStretchBend`((self, particle1, particle2, ...) | Add a stretch-bend term to the force field. |
    | `getForceGroup`((self) -> int) | Get the force group this Force belongs to. |
    | `getNumStretchBends`((self) -> int) | Get the number of stretch-bend terms in the potential function |
    | `getStretchBendParameters`(self, index) | Get the force field parameters for a stretch-bend term. |
    | `setForceGroup`(self, group) | Set the force group this Force belongs to. |
    | `setStretchBendParameters`(self, index, ...) | Set the force field parameters for a stretch-bend term. |
    | `updateParametersInContext`(self, context) | Update the per-stretch-bend term parameters in a Context to match those stored in this Force object. |
    | `usesPeriodicBoundaryConditions`((self) -> bool) | Returns whether or not this force makes use of periodic boundary conditions. |

    `getNumStretchBends`(*self*) → int¶
    :   Get the number of stretch-bend terms in the potential function

    `addStretchBend`(*self*, *particle1*, *particle2*, *particle3*, *lengthAB*, *lengthCB*, *angle*, *k1*, *k2*) → int¶
    :   Add a stretch-bend term to the force field.

        |  |  |
        | --- | --- |
        | Parameters: | - **particle1** (*int*) – the index of the first particle connected by the stretch-bend - **particle2** (*int*) – the index of the second particle connected by the stretch-bend - **particle3** (*int*) – the index of the third particle connected by the stretch-bend - **lengthAB** (*double*) – the equilibrium length of the stretch-bend in bond ab [particle1, particle2], measured in nm - **lengthCB** (*double*) – the equilibrium length of the stretch-bend in bond cb [particle3, particle2], measured in nm - **angle** (*double*) – the equilibrium angle in radians - **k1** (*double*) – the force constant of the product of bond ab and angle a-b-c - **k2** (*double*) – the force constant of the product of bond bc and angle a-b-c (optional, default is the same as k1) |
        | Returns: | the index of the stretch-bend that was added |
        | Return type: | int |

    `getStretchBendParameters`(*self*, *index*)¶
    :   Get the force field parameters for a stretch-bend term.

        |  |  |
        | --- | --- |
        | Parameters: | **index** (*int*) – the index of the stretch-bend for which to get parameters |
        | Returns: | - **particle1** (*int*) –   the index of the first particle connected by the stretch-bend - **particle2** (*int*) –   the index of the second particle connected by the stretch-bend - **particle3** (*int*) –   the index of the third particle connected by the stretch-bend - **lengthAB** (*double*) –   the equilibrium length of the stretch-bend in bond ab [particle1, particle2], measured in nm - **lengthCB** (*double*) –   the equilibrium length of the stretch-bend in bond cb [particle3, particle2], measured in nm - **angle** (*double*) –   the equilibrium angle in radians - **k1** (*double*) –   the force constant of the product of bond ab and angle a-b-c - **k2** (*double*) –   the force constant of the product of bond bc and angle a-b-c |

    `setStretchBendParameters`(*self*, *index*, *particle1*, *particle2*, *particle3*, *lengthAB*, *lengthCB*, *angle*, *k1*, *k2*)¶
    :   Set the force field parameters for a stretch-bend term.

        |  |  |
        | --- | --- |
        | Parameters: | - **index** (*int*) – the index of the stretch-bend for which to set parameters - **particle1** (*int*) – the index of the first particle connected by the stretch-bend - **particle2** (*int*) – the index of the second particle connected by the stretch-bend - **particle3** (*int*) – the index of the third particle connected by the stretch-bend - **lengthAB** (*double*) – the equilibrium length of the stretch-bend in bond ab [particle1, particle2], measured in nm - **lengthCB** (*double*) – the equilibrium length of the stretch-bend in bond cb [particle3, particle2], measured in nm - **angle** (*double*) – the equilibrium angle in radians - **k1** (*double*) – the force constant of the product of bond ab and angle a-b-c - **k2** (*double*) – the force constant of the product of bond bc and angle a-b-c (optional, default is the same as k1) |

    `updateParametersInContext`(*self*, *context*)¶
    :   Update the per-stretch-bend term parameters in a Context to match those stored in this Force object. This method provides an efficient method to update certain parameters in an existing Context without needing to reinitialize it. Simply call setStretchBendParameters() to modify this object’s parameters, then call updateParametersInContext() to copy them over to the Context.

        The only information this method updates is the values of per-stretch-bend term parameters. The set of particles involved in a term cannot be changed, nor can new terms be added.

    `usesPeriodicBoundaryConditions`(*self*) → bool¶
    :   Returns whether or not this force makes use of periodic boundary conditions.

        |  |  |
        | --- | --- |
        | Returns: | true if nonbondedMethod uses PBC and false otherwise |
        | Return type: | bool |

    `__delattr__`¶
    :   x.\_\_delattr\_\_(‘name’) <==> del x.name

    `__format__`()¶
    :   default object formatter

    `__getattribute__`¶
    :   x.\_\_getattribute\_\_(‘name’) <==> x.name

    `__hash__`¶

    `__reduce__`()¶
    :   helper for pickle

    `__reduce_ex__`()¶
    :   helper for pickle

    `__sizeof__`() → int¶
    :   size of object in memory, in bytes

    `__str__`¶

    `getForceGroup`(*self*) → int¶
    :   Get the force group this Force belongs to.

    `setForceGroup`(*self*, *group*)¶
    :   Set the force group this Force belongs to.

        |  |  |
        | --- | --- |
        | Parameters: | **group** (*int*) – the group index. Legal values are between 0 and 31 (inclusive). |

High performance molecular simulation on GPUs


### Navigation

- Application Layer
- Library Layer
  - Core Objects
  - Forces
  - Integrators
  - Extras

©2015, Stanford University and the Authors.
|
Powered by Sphinx 1.3.1
& Alabaster 0.7.7
|
Page source
